# Supplementary material for: Transcriptome analysis of colored calla lily (Zantedeschia rehmannii Engl.) by Illumina sequencing: de novo assembly, annotation and EST-SSR marker development
Source: PeerJ. 2016 Sep 1;4:e2378. doi: 10.7717/peerj.2378 (PMC5012260; doi:10.7717/peerj.2378)
Supplement: Table S1 [file peerj-04-2378-s002.docx]

**Table S1 The 21 accessions of colored calla lily used in present study for transcriptome sequencing and EST-SSR markers development.**

| **Cultivars/hybrids** | **Origins** | **Spathe Color** | **Type** | **Photographs** |
| --- | --- | --- | --- | --- |
| Rehmannii | Netherlands | Pink | Pot-flower | *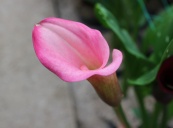* |
| Elmaro | New Zealand | Yellow | Pot-/Cut- flower | *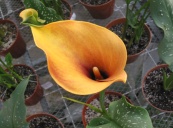* |
| Parfait | American | Pink | Pot-flower | 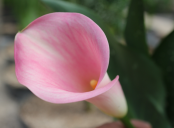 |
| Pink Diamond | New Zealand | Pink | Cut-flower | 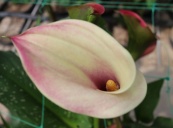 |
| Super Gem | American | Pink | Pot-flower | 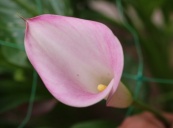 |
| Rose Gem | American | Pink | Pot-flower | *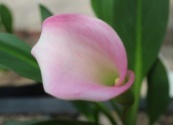* |
| Butter Gold | New Zealand | Yellow | Cut-flower | 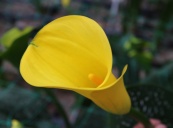 |
| Odessa | Netherlands | Purple | Pot-flower | 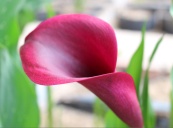 |
| Black Magic | New Zealand | Yellow | Cut-flower | *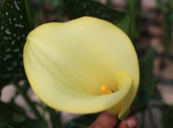* |
| Jingcai Yangguang | China | Yellow | Cut-flower | *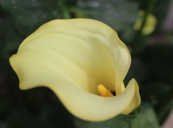* |
| Majestic Red | New Zealand | Red | Pot-flower | 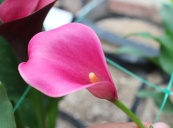 |
| Xiangyuan Red | China | Red | Pot-flower | 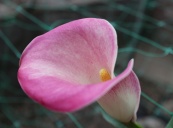 |
| Sunny Baby | New Zealand | Yellow | Pot-/Cut- flower | 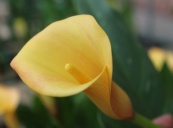 |
| Captain Reno | Netherlands | Purple | Pot-/Cut- flower | 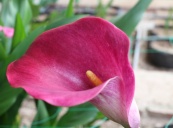 |
| Allure | Netherlands | Red | Pot-flower | 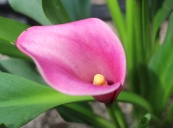 |
| Greta | New Zealand | Purple | Pot-flower | 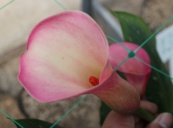 |
| Goldilocks | New Zealand | Yellow | Pot-flower | 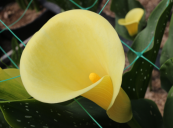 |
| Captain Romance | Netherlands | Pink | Pot-/Cut- flower | 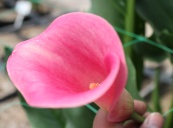 |
| Captain Murano | Netherlands | Orange | Pot-/Cut- flower | 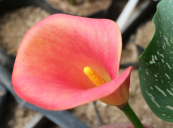 |
| Gold affair | New Zealand | Yellow | Pot-/Cut- flower | 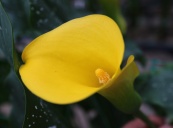 |
| ZH | China | Yellow | Cut-flower | 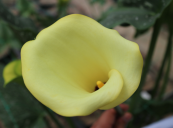 |
